# An Indoor UAV Localization Framework with ESKF Tightly-Coupled Fusion and Multi-Epoch UWB Outlier Rejection

**DOI:** 10.3390/s25247673

**Published:** 2025-12-18

**Authors:** Jianmin Zhao, Zhongliang Deng, Enwen Hu, Wenju Su, Boyang Lou, Yanxu Liu

**Affiliations:** 1 School of Electronic Engineering, Beijing University of Posts and Telecommunications, Beijing 100876, China; teresateng@bupt.edu.cn (J.Z.); dengzhl@bupt.edu.cn (Z.D.); suwj@bupt.edu.cn (W.S.); woshiluobodan@bupt.edu.cn (B.L.); 2 School of Computer and Information, Dezhou University, Dezhou 253023, China; liuyanxu@dzu.edu.cn

**Keywords:** UWB, IMU, NLOS, outlier rejection, ESKF, indoor UAV localization

## Abstract

Unmanned aerial vehicles (UAVs) are increasingly used indoors for inspection, security, and emergency tasks. Achieving accurate and robust localization under Global Navigation Satellite System (GNSS) unavailability and obstacle occlusions is therefore a critical challenge. Due to their inherent physical limitations, Inertial Measurement Unit (IMU)–based localization errors accumulate over time, Ultra-Wideband (UWB) measurements suffer from systematic biases in Non-Line-of-Sight (NLOS) environments and Visual–Inertial Odometry (VIO) depends heavily on environmental features, making it susceptible to long-term drift. We propose a tightly coupled fusion framework based on the Error-State Kalman Filter (ESKF). Using an IMU motion model for prediction, the method incorporates raw UWB ranges, VIO relative poses, and TFmini altitude in the update step. To suppress abnormal UWB measurements, a multi-epoch outlier rejection method constrained by VIO is developed, which can robustly eliminate NLOS range measurements and effectively mitigate the influence of outliers on observation updates. This framework improves both observation quality and fusion stability. We validate the proposed method on a real-world platform in an underground parking garage. Experimental results demonstrate that, in complex indoor environments, the proposed approach exhibits significant advantages over existing algorithms, achieving higher localization accuracy and robustness while effectively suppressing UWB NLOS errors as well as IMU and VIO drift.

## 1. Introduction

In recent years, UAV technology has achieved remarkable progress, and its applications have expanded from traditional military reconnaissance to various civilian domains such as logistics delivery, building inspection, security surveillance, and disaster rescue [1]. These emerging application scenarios impose increasingly stringent requirements on the autonomous navigation and localization capabilities of UAVs. The integrated navigation system composed of GNSS and IMU can achieve centimeter-level positioning accuracy in outdoor environments. In contrast to the vast outdoor environment, indoor spaces lack GNSS signals, preventing UAVs from relying on conventional GNSS-based localization [2]. Although motion capture systems can provide sub-millimeter-level positioning accuracy, their high cost and limited coverage make them unsuitable for practical UAV navigation tasks. Therefore, achieving high-precision and high-robustness autonomous localization for UAVs in complex indoor environments has become a key challenge in the current development of UAV technology [3].

To address the challenges of indoor UAV localization, researchers have proposed various technical solutions, including positioning methods based on Wi-Fi, Bluetooth, vision, IMU, and UWB sensors [4,5,6]. Each of these methods possesses both advantages and limitations, making it difficult to independently provide stable and reliable localization results in complex and dynamic indoor environments. Specifically, Wi-Fi and Bluetooth-based positioning systems have limited accuracy and are easily affected by multipath effects [7,8,9]. Wi-Fi localization approaches exploit physical-layer channel state information (CSI) to achieve high-resolution positioning by jointly estimating multipath parameters in the angle-delay domain. Typical methods include maximum likelihood joint AoA/ToA estimation, variants such as JADED-RIP (Joint Angle and Delay Estimator and Detector), and sparse-recovery schemes based on iterative variational Bayes, which reconstruct a sparse path-gain vector on a discretized angle-delay grid [10,11,12,13,14]. However, these Wi-Fi CSI-based techniques generally require carefully calibrated antenna arrays, accurate synchronization, and favorable propagation conditions, and their performance may deteriorate in cluttered indoor environments with rich multipath. Visual odometry estimates UAV poses by matching feature points between consecutive image frames captured by a camera. It performs well in texture-rich environments; however, its performance deteriorates significantly under severe illumination changes, motion blur, or large textureless areas, potentially leading to localization failure [15,16,17]. The IMU estimates UAV poses by integrating acceleration and angular velocity, offering high-frequency measurements and short-term accuracy, but its localization error accumulates over time, resulting in severe drift [18]. UWB measures distance by calculating the Time of Flight (TOF) of radio signals. Under ideal Line-of-Sight (LOS) conditions, it can provide centimeter-level ranging accuracy. However, its major drawback lies in its extreme sensitivity to NLOS propagation. When signals are obstructed by obstacles, the measured distance tends to be significantly overestimated, severely degrading localization accuracy and reliability [19,20,21].

In indoor settings where NLOS propagation and multipath induce abnormal UWB ranges, researchers have developed a variety of outlier rejection techniques. Statistical methods based on multi-epoch data are simple and effective, as they identify and remove outliers that deviate significantly from the normal distribution through statistical analysis of consecutive measurements. These methods exploit the temporal consistency of data, achieving robust outlier handling with relatively low computational cost. Zhang et al. [22] proposed a multi-epoch outlier rejection algorithm based on Random Sample Consensus (RANSAC) to preprocess UWB measurements, effectively mitigating the negative impact of UWB outliers on the localization accuracy of multi-sensor fusion systems. Fan et al. [23] introduced a sliding-window-based outlier detection method that compares the distance variations between UWB and IMU prediction windows against a predefined threshold to detect abnormal values. However, this approach is highly sensitive to the chosen threshold and requires manual tuning for different environments. Another effective strategy is to directly handle outliers during the filtering process. Li et al. [24] proposed a method based on Mahalanobis distance and chi-square testing, in which the variance of UWB measurements associated with outliers is enlarged to reduce their influence on the filter’s state update. However, when the proportion of outliers is high, the effectiveness of this method becomes limited. Additionally, another straightforward approach is to compare the Mahalanobis distance against a predefined threshold and directly discard abnormal measurements [25]. Beyond processing measurement data itself, some studies attempt to detect and reject outliers by analyzing the physical characteristics of the received signals. Wang et al. [26] exploited the fact that received signal power attenuation in NLOS paths is typically greater than in LOS paths, using the power difference as a signal gain indicator and comparing it with a threshold to identify and eliminate NLOS measurements. However, in complex signal environments, this method may lead to misclassification. Stahlke et al. [27] directly fed the UWB Channel Impulse Response (CIR) into a Convolutional Neural Network (CNN) to classify LOS/NLOS conditions, thereby improving classification reliability and localization robustness in complex indoor environments. Pei et al. [28] proposed FCN-Attention, which combines a Fully Convolutional Network with an attention mechanism. Wang et al. [29] used CIR waveform features as inputs to systematically compare traditional machine learning models such as SVM, MLP, KNN, and XGBoost. However, the performance of these methods typically depends on the diversity and coverage of training data. Cross-environment generalization and online deployment remain challenging.

Although UWB systems can achieve high-precision 3D localization, their relatively low measurement frequency and sensitivity to signal occlusion limit their ability to provide continuous positioning during high-dynamic UAV flights. In contrast, Inertial Navigation Systems (INS) have the advantages of independence from external infrastructure and high output frequency, making them widely employed in indoor navigation tasks [30]. However, since INS relies on integration operations, its errors accumulate rapidly over time. To overcome the inherent limitations of single-sensor systems, researchers commonly integrate UWB with INS to achieve robust, high-frequency, and long-term stable localization [31]. Typical fusion strategies include filtering-based and optimization-based methods. Among the filtering-based fusion algorithms, the Extended Kalman Filter (EKF) is one of the most widely used techniques. For example, Liu et al. [32] proposed a UWB/IMU data fusion method based on EKF, which adopts a traditional linear regression calibration model and a mean filter for distance smoothing, effectively improving the system’s localization accuracy and robustness. Similarly, Feng et al. [33] utilized EKF to fuse IMU and UWB data and introduced both constant-velocity and constant-acceleration motion models to achieve smoother position estimation, thereby further enhancing system robustness and localization accuracy. However, the EKF is essentially an extension of the standard Kalman Filter (KF), where the nonlinear observation equations are linearized via first-order Taylor expansion for state estimation. When the system exhibits strong nonlinearity, this approximation neglects higher-order terms, leading to a degradation in estimation accuracy. To address the limitations of EKF in highly nonlinear systems, You et al. [34] employed the Unscented Kalman Filter (UKF) for IMU and UWB fusion localization. The UKF uses the unscented transform to more accurately propagate the mean and covariance of the system states, avoiding the linearization errors present in EKF and thus achieving higher localization accuracy. Both the Cubature Kalman Filter (CKF) and the UKF are designed to address the accuracy limitations of EKF when dealing with strongly nonlinear systems. Their core principle lies in using a set of carefully selected sigma points to propagate the mean and covariance through nonlinear transformations. Ji et al. [30] implemented UWB/IMU fusion using CKF, incorporating an adaptive factor to adjust the measurement noise covariance matrix and introducing a fading factor to suppress filter divergence.However, for INS systems where attitude is represented on Lie groups, both UKF and CKF still face challenges in computational efficiency and stability when handling attitude manifold nonlinearity. Consequently, the ESKF has gained widespread attention due to its effective handling of attitude error. This framework linearizes only the small error states, significantly enhancing the robustness and accuracy of the filter. Marković et al. [35] proposed a multi-sensor fusion algorithm based on ESKF, which integrates measurements from IMU, UWB, camera, and LiDAR while considering sensor drift and calibration errors. Moreover, an arbitration mechanism was introduced to remove abnormal sensor measurements before the fusion stage.

In addition to filtering-based multi-sensor fusion algorithms, optimization-based methods have increasingly attracted attention. Zheng et al. [36] proposed a tightly coupled graph optimization method for fusing UWB and IMU data. This model effectively utilizes multiple UWB tags, providing a new direction for the practical application of UWB/IMU fusion techniques. Xu et al. [37] proposed a general graph optimization-based localization framework that introduces ranging constraints and trajectory smoothness constraints into the position graph. The framework models and estimates the robot’s trajectory within a sliding window and solves for the optimal poses using optimization algorithms. Kang et al. [38] addressed the accuracy limitations in UWB-assisted UAV localization by proposing a factor graph fusion method based on incremental smoothing. The study aims to overcome the limitation of existing approaches that treat individual UWB range measurements as weak constraints. The core idea is to integrate high-frequency single-point UWB ranging data, low-frequency multilateration results, and IMU measurements into a unified factor graph framework. This method significantly improves localization accuracy and system robustness, especially in scenarios involving large motion variations. Song et al. [39] proposed a tightly coupled UWB/INS navigation system based on factor graph optimization to enhance UAV localization accuracy and robustness in indoor environments. The key innovation of this approach lies in discarding the traditional loosely coupled or semi-tightly coupled frameworks. Instead, it directly fuses raw UWB ranging information and IMU preintegration data within a factor graph, jointly optimizing all state variables, including position, velocity, attitude, and IMU biases. This tightly coupled architecture fully leverages the complementary characteristics of UWB and INS, effectively suppressing interference caused by UWB multipath effects. Zheng et al. [40] proposed a tightly coupled factor graph optimization method integrating UWB and LiDAR to address UWB localization drift caused by NLOS effects in indoor environments. The method fuses multi-source observation data within the factor graph and introduces an NLOS detection and correction module to identify and compensate for invalid UWB range measurements. Although optimization-based methods offer clear advantages in modeling flexibility and fusion accuracy, they still face certain limitations in practical applications. First, the optimization process is sensitive to the initial state; in dynamic scenarios or with large initial errors, it tends to settle in local minima and may even fail to converge. Second, optimization-based methods typically incur high computational complexity, making it difficult to meet the real-time localization requirements of highly dynamic platforms such as UAVs.

To address the limitations of existing methods and the aforementioned challenges, this paper proposes a high-precision multi-sensor fusion localization approach for indoor UAVs. The proposed method adopts a tightly coupled architecture based on the ESKF, which fuses multi-source information (including IMU prior propagation, raw UWB ranging, VIO relative poses, and TFmini altitude observations) within a unified state space to achieve efficient joint state updates and enhance system robustness. The main contributions of this work are summarized as follows:

- A tightly coupled fusion framework built upon the ESKF leverages IMU, VIO, UWB, and TFmini observations to achieve accurate and robust localization. This framework fully exploits the complementary advantages of different sensors to achieve high-accuracy and high-robustness localization performance.
- A sliding-window UWB model referenced to short-term VIO poses employs a RANSAC-based multi-epoch consistency check to reject outliers. This method significantly improves the statistical consistency and reliability of UWB observations, mitigates the influence of outliers on measurement updates, and thereby enhances fusion stability and positioning accuracy.
- Extensive field experiments were conducted on a UAV system platform to validate the proposed algorithm in an underground parking garage. The experimental results demonstrate that the proposed method significantly outperforms single-sensor localization approaches in both accuracy and robustness, providing a reliable technical foundation for autonomous indoor navigation of UAV.

The remainder of this paper is organized as follows: Section 2 details the proposed VIO-constrained multi-epoch outlier rejection method and the ESKF-based multi-sensor fusion algorithm. Section 3 presents and analyzes field experimental results to validate the effectiveness of the proposed approach. Section 4 concludes the paper.

## 2. Methods

**Figure 1:**
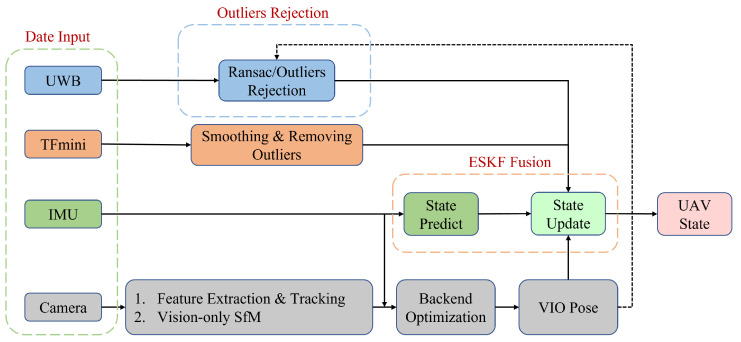
System overview of the proposed localization system.

This study builds a multi-source heterogeneous sensor fusion localization system on a UAV platform, integrating an IMU, a UWB tag, a stereo camera, a 3D LiDAR, and a TFmini. A single UWB tag receives wireless ranging signals from *N* UWB anchors deployed indoors, whose global positions are known (denoted as $A_{i},\;i=1,2,\ldots ,N$, with 3D coordinates $p_{A_{i}}^{w}\in R^{3}$ in the world frame). The world coordinate frame *w* is defined with respect to the UWB anchor layout. Specifically, the origin is located at the projection of anchor $A_{1}$ on the ground. The *x*-axis points towards the projection of anchor $A_{2}$, the *z*-axis is vertically upward, and the *y*-axis is determined by the right-hand rule. The tightly coupled system efficiently fuses four heterogeneous data sources: IMU, UWB, VIO, and TFmini. The core of the proposed system comprises two components: a VIO-constrained multi-epoch outlier rejection algorithm, and an ESKF-based tightly coupled fusion localization algorithm. The VIO-constrained outlier rejection leverages VIO’s short-horizon reliable pose estimates to identify and suppress NLOS and other abnormal UWB ranges in real time, substantially improving the usability of UWB observations and the overall accuracy of the fused system. The ESKF-based tightly coupled fusion algorithm effectively integrates measurements of different rates and characteristics to achieve accurate and robust state estimation. To clearly illustrate the information flow and fusion mechanism of the entire system, the overall architecture of the proposed tightly coupled fusion localization system is shown in Figure 1.

### 2.1. VIO-Constrained Multi-Epoch Outlier Rejection Algorithm

**Figure 2:**
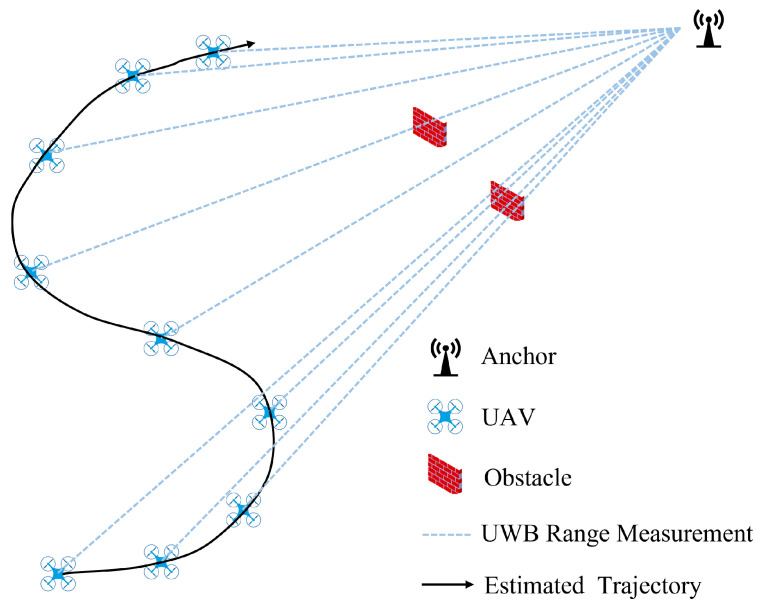
Schematic diagram of the outlier rejection algorithm model.

RANSAC is a general-purpose robust parameter estimation method that effectively handles significant outliers that may exist in UWB observation data. As a resampling technique, RANSAC first generates candidate solutions using the minimum number of observations required for the estimation model, and then gradually expands and incorporates consistent data points that satisfy the model constraints. To detect and reject outliers, we calculate the residuals between the predicted geometric distances (from the VIO trajectory and UWB anchor locations) and the measured UWB distances. If a residual exceeds a predefined threshold, indicating inconsistency with the model, the corresponding measurement is flagged as an outlier. In this paper, an improved RANSAC-based algorithm is employed to robustly reject UWB ranging outliers. The core idea of the algorithm is to leverage the high-accuracy relative trajectory information provided by VIO over a short period to perform consistency checks on multi-epoch UWB ranging data, thereby accurately identifying and eliminating outliers. The design and implementation of the algorithm primarily involve two aspects: the construction of the outlier rejection model and the specific implementation process. Figure 2 illustrates the conceptual diagram of the proposed RANSAC outlier rejection algorithm model.

#### 2.1.1. Outlier Rejection Algorithm Model

In a short period of time, VIO can provide a relative trajectory that is highly consistent with the true trajectory. Therefore, we can use this trajectory and all corresponding UWB ranging data to estimate the relative positions of the UWB anchors, and use this as a model to identify outliers. In this paper, we employ a nonlinear optimization method to solve the above model and determine the positions of the anchors. For the *i*-th UWB anchor, assuming there are *Q* ranging data points within the sliding window, we construct an objective function to minimize the sum of squared measurement residuals:

(1) $$F\left(p_{A_{i}}^{w}\right)=\min_{p_{A_{i}}^{w}}\sum_{j=1}^{Q}{\left({∥p_{b_{j}}^{w}+R_{b_{j}}^{w}p_{tag}^{b}-p_{A_{i}}^{w}∥}_{2}-d_{i,j}\right)}^{2}$$

Here, $p_{A_{i}}^{w}$ denotes the model-estimated position of the *i*-th anchor in the world frame. $p_{b_{j}}^{w}$ and $R_{b_{j}}^{w}$ are, respectively, the position and orientation of the UAV in the world frame at the *j*-th UWB ranging epoch, obtained from VIO after coordinate transformation. Accordingly, the positions and orientations corresponding to all ranging data within the sliding window are represented as $T=\{\left(R_{b_{1}}^{w},p_{b_{1}}^{w}\right),\left(R_{b_{2}}^{w},p_{b_{2}}^{w}\right),\cdots ,\left(R_{b_{Q}}^{w},p_{b_{Q}}^{w}\right)\}$. $p_{\mathrm{tag}}^{b}$ is the pre-calibrated extrinsic of the UWB tag with respect to the UAV. $d_{i,j}$ is the range measurement from the UWB tag to the *i*-th anchor at the *j*-th epoch; correspondingly, all range measurements of the *i*-th anchor within the sliding window are denoted as $D_{i}=\left\{d_{i,1},d_{i,2},\cdots ,d_{i,Q}\right\}$.

This paper employs the Levenberg–Marquardt (LM) algorithm to solve this nonlinear least-squares problem. To achieve fast convergence, we compute the Jacobian of the objective function with respect to the anchor position. The residual $r_{i,j}$ is defined as:

(2) $$r_{i,j}={∥p_{b_{j}}^{w}+R_{b_{j}}^{w}p_{tag}^{b}-p_{A_{i}}^{w}∥}_{2}-d_{i,j}$$

The Jacobian matrix $J_{j}$ is:

(3) $$J_{j}=\frac{\partial r_{i,j}}{\partial p_{A_{i}}^{w}}=\frac{{\left(p_{A_{i}}^{w}-\left(p_{b_{j}}^{w}+R_{b_{j}}^{w}p_{tag}^{b}\right)\right)}^{T}}{{∥p_{b_{j}}^{w}+R_{b_{j}}^{w}p_{tag}^{b}-p_{A_{i}}^{w}∥}_{2}}$$

The Levenberg–Marquardt (LM) algorithm is a nonlinear least-squares solver that combines the Gauss–Newton method and gradient descent. It iteratively approaches the optimal solution. The solving procedure is as follows:

1. **Initialization:** Provide an initial estimate of the anchor position $p_{A_{i}}^{w,0}$.
2. **Iterative update:** At the *k*-th iteration, we seek an increment $\Delta p_{A_{i}}^{w}$ that minimizes the sum of squared residuals. The LM algorithm approximates the nonlinear least-squares problem by a linear system and introduces a damping factor λ to control the step size:
  (4) $$\left(J^{T}J+\lambda I\right)\Delta p_{A_{i}}^{w}=-J^{T}r$$
  where J is the Jacobian evaluated at the current estimate $p_{A_{i}}^{w,k}$, with row vectors $\frac{\partial r_{i,j}}{\partial p_{A_{i}}^{w,k}}$. r is the vector collecting all *Q* ranging residuals. I is the identity matrix. λ is the damping factor that balances between gradient descent and Gauss–Newton updates.
3. **Update and convergence check:** Solve the linear system above to obtain $\Delta p_{A_{i}}^{w}$, then update the anchor position estimate:
  (5) $$p_{A_{i}}^{w,k+1}=p_{A_{i}}^{w,k}+\Delta p_{A_{i}}^{w}$$
  If the new estimate decreases the objective function *F*, accept the update and decrease the damping factor λ; otherwise, reject the update and increase λ. This process repeats until a predefined convergence criterion is met (the norm of $\Delta p_{A_{i}}^{w}$ falls below a threshold, or the maximum number of iterations is reached). Finally, we obtain an optimal estimate of the anchor position.

#### 2.1.2. Outlier Rejection Procedure

To effectively remove outliers in UWB ranging data, we propose a VIO-constrained multi-epoch outlier rejection algorithm in which RANSAC serves as a key component. The entire algorithm is carried out by combining the relative poses provided by the UAV’s VIO with the UWB ranging data. First, we set the maximum number of RANSAC iterations $N_{iter}$, the minimum sample size *P* for model construction, the residual threshold ϵ, and the inlier count threshold *L*. In each iteration, we randomly select *P* ranging samples from the UWB sliding window and, together with the VIO trajectory, estimate an initial UWB anchor position using the LM algorithm. This yields the current model. Next, we evaluate all remaining ranging measurements against this model by computing their residuals and the residual sum. A measurement is marked as an inlier if its residual is below the preset threshold ϵ. After multiple iterations, we select the model with the smallest residual sum and regard the associated set of ranging data as valid, since these measurements are most likely to originate from LOS propagation. Finally, all measurements classified as outliers are discarded, and only the valid data are used in the subsequent ESKF update. This procedure effectively increases the reliability of UWB observations and underpins the robustness and accuracy improvements of the proposed fusion framework. The pseudocode of the proposed UWB outlier rejection algorithm is provided in Algorithm 1.
**Algorithm 1** VIO-Constrained Multi-epoch Outlier Rejection**Input:** UWB ranging data set within the sliding window $D_{i}$, UAV poses estimated by VIO T **Input:** RANSAC iteration number $N_{iter}$, minimum sample size *P*, ranging residual threshold ϵ, minimum inlier count *L* **Output:** Valid UWB ranging data set identified as inliers $I_{final}$  1:k←0, $I_{final}\leftarrow \emptyset$, $\epsilon_{\mathrm{optimal}}\leftarrow \infty$  2:**for** k←1 to $N_{iter}$ **do**  3:     $D_{sample}\leftarrow$ Randomly select *P* samples from $D_{i}$  4:     $p_{A_{i}}^{\mathrm{model}}\leftarrow$ Solve anchor position with LM using $\left(D_{sample},T\right)$  5:     $I_{\mathrm{temp}}\leftarrow \emptyset$  6:     **for** each measurement $m\in D_{i}$ **do**  7:         r← Calculate residual $\left(m,p_{A_{i}}^{\mathrm{model}},T\right)$  8:         $\epsilon_{\mathrm{better}}\leftarrow$ the sum of *r*  9:         **if** r<ϵ **then**10:            $I_{\mathrm{temp}}\leftarrow I_{\mathrm{temp}}\cup \left\{m\right\}$11:         **end if**12:     **end for**13:     **if** $|I_{\mathrm{temp}}|>L$ **then**14:         **if** $\epsilon_{\mathrm{better}}<\epsilon_{\mathrm{optimal}}$ **then**15:            $\epsilon_{\mathrm{optimal}}\leftarrow \epsilon_{\mathrm{better}}$16:            $I_{final}\leftarrow I_{\mathrm{temp}}$17:         **end if**18:     **end if**19:**end for**20:**return** $I_{final}$

### 2.2. ESKF-Based Multi-Sensor Fusion Localization Algorithm

By linearizing the error state on the Lie algebra, the ESKF significantly reduces the impact of nonlinear effects on filtering performance while maintaining low computational complexity, making it suitable for real-time UAV localization. Although the UWB system can provide position measurements of the UAV, accurate attitude perception during flight is equally crucial for precise localization. The IMU supplies high-frequency angular velocity and acceleration measurements, capturing rapid dynamic changes of the UAV; however, its measurement errors accumulate over time, leading to drift. VIO provides relative pose estimates using visual information but is prone to failure in environments with poor texture or severe illumination variations. The TFmini offers stable altitude information, compensating for VIO’s insufficient accuracy along the vertical axis. To efficiently fuse multi-source observation information, this paper proposes a tightly coupled ESKF (TC-ESKF) algorithm that integrates measurements from IMU, UWB, VIO, and TFmini to achieve accurate state estimation and error correction.

#### 2.2.1. State Definition and Error Modeling

Within the ESKF framework, the system state is partitioned into the nominal state, the error state, and the true state. The nominal state evolves on the manifold according to the nonlinear dynamics (position and velocity lie in Euclidean space, while attitude lies on SO(3)); the error state is modeled in the tangent space of the manifold and is kept small to enable first-order linearization and Kalman updates; the true state is obtained by composing the nominal state with the error state, where position and velocity use additive composition, and attitude uses left-multiplication of quaternions. This paper represents attitude with quaternions, models the attitude error by a 3D small-angle vector δθ, and enforces quaternion normalization and error reset after error injection to ensure numerical stability. The reference frames are defined as follows: position p, velocity v, and gravity g are expressed in the world frame *w*; the attitude quaternion q denotes the rotation from the body frame *b* to the world frame *w* (with R(q) the rotation matrix corresponding to q); the gyroscope and accelerometer biases $b_{g}$ and $b_{a}$ are defined in the body frame. Based on these conventions, the nominal state vector is defined as

(6) $$x={\left[\begin{array}{ccccc} p & v & q & b_{a} & b_{g} \end{array}\right]}^{⊤}\in R^{16}$$

where $p\in R^{3}$ is position, $v\in R^{3}$ is velocity, q∈H is the attitude quaternion, and $b_{a},b_{g}\in R^{3}$ are the accelerometer and gyroscope biases, respectively.

The corresponding error state vector is

(7) $$\delta x={\left[\begin{array}{ccccc} \delta p & \delta v & \delta \theta & \delta b_{a} & \delta b_{g} \end{array}\right]}^{⊤}\in R^{15}$$

where the attitude error is represented by a small-angle rotation vector δθ. This vector is converted into an error quaternion δq to be composed with the nominal quaternion. The first-order approximation of δq is $\delta q\approx {\left[\begin{array}{cc} 1 & {\left(\delta \theta /2\right)}^{⊤} \end{array}\right]}^{⊤}$. The true state is described by the composition of the nominal and error states, with the relationship

(8) $$x^{t}=\left[\begin{array}{c} p+\delta p\\ v+\delta v\\ q⊗\delta q\\ b_{a}+\delta b_{a}\\ b_{g}+\delta b_{g} \end{array}\right]$$

where ⊗ denotes quaternion multiplication. This modeling approach enables the filter to maintain high numerical stability under nonlinear conditions.

#### 2.2.2. IMU Motion Model and State Propagation

The IMU measures angular velocity $\omega_{m}$ and specific force $a_{m}$ through the gyroscope and accelerometer, respectively. These measurements are affected by biases and random noise. The IMU motion model is given in (9):

(9) $$\omega_{m}=\omega +b_{g}+n_{g},\;a_{m}=R{\left(q\right)}^{⊤}\left(a-g\right)+b_{a}+n_{a}$$

Here, $n_{a}$ and $n_{g}$ denote the accelerometer and gyroscope measurement noises, both modeled as zero-mean Gaussian distributions $n_{a}∼N\left(0,\sigma_{a}^{2}\;\cdot \;I_{3\times 3}\right)$ and $n_{g}∼N\left(0,\sigma_{g}^{2}\;\cdot \;I_{3\times 3}\right)$. The accelerometer and gyroscope biases, $b_{a}$ and $b_{g}$, follow random-walk processes, therefore, ${\dot{b}}_{g}=n_{bg}$ and ${\dot{b}}_{a}=n_{ba}$, where $n_{bg}∼N\left(0,\sigma_{bg}^{2}\;\cdot \;I_{3\times 3}\right)$ and $n_{ba}∼N\left(0,\sigma_{ba}^{2}\;\cdot \;I_{3\times 3}\right)$. The gravity vector is $g={\left[0,0,-9.81\right]}^{⊤}$. Whenever new IMU measurements arrive, they are used to update the nominal state. Consequently, the continuous-time propagation of the nominal state is

(10) $$\begin{array}{cc} \dot{p} & =v \end{array}$$

(11) $$\begin{array}{cc} \dot{v} & =R\left(q\right)\left(a_{m}-b_{a}\right)+g \end{array}$$

(12) $$\begin{array}{cc} \dot{q} & =\frac{1}{2}\Omega \left(\omega_{m}-b_{g}\right)q \end{array}$$

(13) $$\begin{array}{cc} {\dot{b}}_{a} & =0,\;{\dot{b}}_{g}=0 \end{array}$$

where

(14) $$\Omega \left(\omega \right)=\left[\begin{array}{cc} 0 & -\omega^{⊤}\\ \omega & -{\left[\omega \right]}_{\times } \end{array}\right],\;{\left[\omega \right]}_{\times }=\left[\begin{array}{ccc} 0 & -\omega_{z} & \omega_{y}\\ \omega_{z} & 0 & -\omega_{x}\\ -\omega_{y} & \omega_{x} & 0 \end{array}\right].$$

The true state and the nominal state satisfy the same dynamic model; the difference is that the true state includes sensor noise terms, whereas the nominal state is driven only by ideal, noise-free measurements. By comparing the evolution of the true and nominal states, we obtain the continuous-time error-state equations. By performing first-order linearization and discretization of the error-state equations, we arrive at the discrete-time error-state propagation model as shown in (15):

(15) $$\delta x_{k+1}=\left[\begin{array}{c} \delta p_{k+1}\\ \delta v_{k+1}\\ \delta \theta_{k+1}\\ \delta b_{a,k+1}\\ \delta b_{g,k+1} \end{array}\right]=\left[\begin{array}{c} \delta p_{k}+\delta v_{k}\Delta t\\ \delta v_{k}+\left(-R\left(q_{b_{k}}^{n}\right){\left[a_{m,k}-b_{a,k}\right]}_{\times }\delta \theta_{k}-R\left(q_{b_{k}}^{n}\right)\;\delta b_{a,k}\right)\Delta t+n_{v,k}\\ R{\left(q_{b_{k}}^{n}\right)}^{⊤}\left\{\left(\omega_{m,k}-b_{g,k}\right)\Delta t+\delta \theta_{k}-\delta b_{g,k}\Delta t+n_{\omega ,k}\right\}\\ \delta b_{a,k}+n_{ba,k}\\ \delta b_{g,k}+n_{bg,k} \end{array}\right]$$

The propagation process includes recursive updates of both the error state and its covariance. The complete discrete-time propagation is therefore given by (16):

(16) $$\begin{array}{cc} \delta x_{k+1}^{-} & =F_{k}\;\delta x_{k}^{+}+G_{k}\;w_{k}\\ P_{k+1}^{-} & =F_{k}\;P_{k}^{+}F_{k}^{⊤}+G_{k}\;Q_{k}\;G_{k}^{⊤} \end{array}$$

where $F_{k}$ is the state transition matrix, $G_{k}$ is the process noise input matrix, $w_{k}$ is the process noise vector, and $Q_{k}$ is the process noise covariance. Their specific forms are given in (17):

(17) $$\begin{array}{cc} F_{k} & =\left[\begin{array}{ccccc} I_{3\times 3} & \Delta t\;\cdot \;I_{3\times 3} & 0 & 0 & 0\\ 0 & I_{3\times 3} & -R\left(q_{b_{k}}^{n}\right){\left[a_{m,k}-b_{a,k}\right]}_{\times }\Delta t & -R(q_{b_{k}}^{n})\Delta t & 0\\ 0 & 0 & R{\left(q_{b_{k}}^{n}\right)}^{⊤}\left((\omega_{m,k}-b_{g,k})\Delta t\right) & 0 & -\Delta t\;\cdot \;I_{3\times 3}\\ 0 & 0 & 0 & I_{3\times 3} & 0\\ 0 & 0 & 0 & 0 & I_{3\times 3} \end{array}\right]\\ G_{k} & =\left[\begin{array}{cccc} 0 & 0 & 0 & 0\\ I_{3\times 3} & 0 & 0 & 0\\ 0 & I_{3\times 3} & 0 & 0\\ 0 & 0 & I_{3\times 3} & 0\\ 0 & 0 & 0 & I_{3\times 3} \end{array}\right]\\ w_{k} & =\left[\begin{array}{c} n_{v,k}\\ n_{\omega ,k}\\ n_{ba,k}\\ n_{bg,k} \end{array}\right]\\ Q_{k} & =\left[\begin{array}{cccc} \sigma_{a}^{2}\Delta t^{2}\;\cdot \;I_{3\times 3} & 0 & 0 & 0\\ 0 & \sigma_{g}^{2}\Delta t^{2}\;\cdot \;I_{3\times 3} & 0 & 0\\ 0 & 0 & \sigma_{ba}^{2}\Delta t\;\cdot \;I_{3\times 3} & 0\\ 0 & 0 & 0 & \sigma_{bg}^{2}\Delta t\;\cdot \;I_{3\times 3} \end{array}\right] \end{array}$$

#### 2.2.3. Multi-Sensor Observation Model

During the measurement update stage, this paper fuses observations from UWB, VIO, and TFmini. For UWB, we directly use raw ranging data as the filter’s measurement input rather than first computing positions and then using them as measurements. This tightly coupled strategy avoids information loss and error magnification caused by intermediate processing. For the camera, the pose provided by VIO is directly used as a measurement to the filter. The TFmini ranging measurement serves as a direct observation of altitude, adding a height constraint that compensates for the limited vertical accuracy of UWB and VIO. By fusing these observations, the filter fully leverages the strengths of each sensor to achieve more accurate and robust state estimation.

Assume that at time *k*, the available observations include *M* UWB ranges $z_{\mathrm{UWB}}={\left[z_{1},z_{2},\ldots ,z_{M}\right]}^{⊤}$, the VIO-provided UAV pose $z_{\mathrm{VIO}}$, and the TFmini range $z_{\mathrm{tf}}$. The relationship between these observations and the system state is described by a nonlinear measurement function h(x). The specific observation models are as follows:

For UWB observations, the localization system comprises several anchors and a UWB tag mounted on the UAV. Let the position and attitude of the body frame *b* in the world frame be $p_{b}^{w}\in R^{3}$ and $R_{b}^{w}\in SO\left(3\right)$, respectively. The UWB tag’s position in the body frame is $t_{u}^{b}\in R^{3}$. Then, the antenna phase center in the world frame is

(18) $$p_{u}^{w}=p_{b}^{w}+R_{b}^{w}t_{u}^{b}.$$

Accordingly, the ideal range between the *i*-th anchor and the UAV-mounted UWB antenna is the Euclidean distance between them:

(19) $${\hat{z}}_{i}=∥p_{u}^{w}-p_{A_{i}}^{w}∥.$$

Accounting for measurement noise, the actual observation is modeled as

(20) $$z_{i}=h_{i}\left(x\right)+n_{i}=∥p_{b}^{w}+R_{b}^{w}t_{u}^{b}-p_{A_{i}}^{w}∥+n_{i},$$

where $h_{i}\left(x\right)$ is the nonlinear measurement function and $n_{i}$ is zero-mean Gaussian noise, i.e., $n_{i}∼N\left(0,\sigma_{\mathrm{uwb}}^{2}\right)$. In practical UWB ranging scenarios, however, the Gaussian assumption can be violated due to NLOS propagation and multipath effects, and several works have reported heavy-tailed or skewed error distributions and advocated non-Gaussian or robust noise models for UWB measurements [41]. For simplicity and clarity of the formulation, we do not adopt such non-Gaussian noise models in this work.

To enable updates within the filtering framework, we linearize the measurement equation. Define

(21) $$d_{i}=p_{u}^{w}-p_{A_{i}}^{w},\;\rho_{i}=∥d_{i}∥,\;{\hat{e}}_{i}=\frac{d_{i}}{\rho_{i}}.$$

Then the UWB residual can be approximated as

(22) $$r_{i}=z_{i}-{\hat{z}}_{i}\approx H_{i}\;\delta x+n_{i},$$

with the measurement Jacobian

(23) $$H_{i}=\left[\begin{array}{cccc} {\hat{e}}_{i}^{⊤} & 0_{1\times 3} & {\hat{e}}_{i}^{⊤}R_{b}^{w}{\left[t_{u}^{b}\right]}_{\times } & 0_{1\times 6} \end{array}\right].$$

Here, ${\hat{e}}_{i}\in R^{3}$ is the unit direction vector from the UAV to the anchor, and ${\left[t_{u}^{b}\right]}_{\times }$ is the skew-symmetric matrix of $t_{u}^{b}$. The Jacobian shows that UWB observations are sensitive to position and attitude errors, but insensitive to velocity and IMU biases.

The VIO output used in this work comes from the open-source project VINS-Fusion [42], which provides the pose of the body frame *b* in the world frame *w*. Within our filtering framework, we use only the VIO-provided position measurement $p_{b,\mathrm{meas}}^{w}$ as a direct constraint on the UAV position.

The VIO position measurement is modeled as

(24) $$z_{\mathrm{vio}}=p_{b,\mathrm{meas}}^{w}=h_{p}\left(x\right)+n_{p},$$

where

(25) $$h_{p}\left(x\right)=p_{b}^{w},$$

and $n_{p}∼N\left(0,\;R_{p,\mathrm{vio}}\right)$ is the position measurement noise.

The position residual is defined as

(26) $$r_{p}=p_{b,\mathrm{meas}}^{w}-{\hat{p}}_{b}^{w},\;{\hat{p}}_{b}^{w}=p_{b}^{w}.$$

Linearizing around the nominal state $\bar{x}$ yields

(27) $$r_{p}\approx H_{\mathrm{VIO}}\;\delta x+n_{p},\;n_{p}∼N\left(0,\;R_{p,\mathrm{vio}}\right).$$

The corresponding measurement Jacobian for the position residual is

(28) $$H_{\mathrm{vio}}=\left[\begin{array}{ccccc} -I & 0 & 0 & 0 & 0 \end{array}\right].$$

From the Jacobian, it is evident that the VIO-provided position information is sensitive only to the position error δp, and is insensitive to velocity, attitude, and IMU biases.

TFmini is a laser range sensor whose measurement can be modeled as the distance, in the sensor coordinate frame, from the sensor optical center along a given ray to the geometric intersection with the ground plane in the world frame. Let *s* denote the TFmini coordinate frame. The extrinsic calibration of TFmini with respect to the body frame is $\left(t_{s}^{b},R_{s}^{b}\right)$. Denote by $u^{s}$ the unit direction vector of the TFmini beam in the sensor frame; its representations in the body and world frames are, respectively,

(29) $$u^{b}=R_{s}^{b}\;u^{s},\;u^{w}=R_{b}^{w}\;u^{b}.$$

The sensor optical center in the world frame is

(30) $$p_{s}^{w}=p_{b}^{w}+R_{b}^{w}\;t_{s}^{b}.$$

Approximate the ground as a plane

(31) $$\Pi :\;n^{⊤}x+d=0,\;∥n∥=1,$$

where n is the plane normal expressed in the world frame and *d* is the plane offset. The ideal TFmini measurement is defined as the signed distance $\hat{z}$ from the optical center $p_{s}^{w}$ along direction $u^{w}$ to the intersection with the plane Π. Thus, the predicted measurement is

(32) $$\hat{z}=\frac{-n^{⊤}p_{s}^{w}-d}{n^{⊤}u^{w}}=\frac{-n^{⊤}\left(p_{b}^{w}+R_{b}^{w}\;t_{s}^{b}\right)-d}{n^{⊤}R_{b}^{w}\;u^{b}}.$$

Let (32) be the nonlinear measurement function $h_{\mathrm{tf}}\left(x\right)$. The TFmini measurement model is then

(33) $$z_{\mathrm{tf}}=h_{\mathrm{tf}}\left(x\right)+n_{\mathrm{tf}},\;n_{\mathrm{tf}}∼N\left(0,\;\sigma_{\mathrm{tf}}^{2}\right),$$

where $\sigma_{\mathrm{tf}}$ depends on surface reflectance, range, and attitude.

Linearizing $h_{\mathrm{tf}}\left(x\right)$ to first order around the nominal state $\bar{x}$, we construct the residual

(34) $$r_{\mathrm{tf}}=z_{\mathrm{tf}}-h_{\mathrm{tf}}\left(\bar{x}\right)\;\approx \;H_{\mathrm{TF}}\;\delta x+n_{\mathrm{tf}}.$$

For Jacobian derivation, define

(35) $$\alpha ≜n^{⊤}u^{w}=n^{⊤}R_{b}^{w}u^{b},\;\beta ≜n^{⊤}p_{s}^{w}+d,$$

so that $\hat{z}=-\beta /\alpha$. Taking derivatives of the main blocks yields

(36) $$\begin{array}{cc} \frac{\partial \hat{z}}{\partial p_{b}^{w}}\; & =-\frac{n^{⊤}}{\alpha }, \end{array}$$

(37) $$\begin{array}{cc} \frac{\partial \hat{z}}{\partial \delta \theta }\; & =-\frac{1}{\alpha }\;n^{⊤}\left(R_{b}^{w}{\left[t_{s}^{b}\right]}_{\times }\right)\;+\;\frac{\beta }{\alpha^{2}}\;n^{⊤}\left(R_{b}^{w}{\left[u^{b}\right]}_{\times }\right), \end{array}$$

(38) $$\begin{array}{cc} \frac{\partial \hat{z}}{\partial v_{b}^{w}} & =0_{1\times 3},\;\frac{\partial \hat{z}}{\partial b_{g}}=0_{1\times 3},\;\frac{\partial \hat{z}}{\partial b_{a}}=0_{1\times 3}. \end{array}$$

Therefore, the TFmini measurement Jacobian is

(39) $$H_{\mathrm{TF}}=\left[\begin{array}{ccccc} -\frac{n^{⊤}}{\alpha }\; & \;0_{1\times 3}\;\; & \left(-\frac{n^{⊤}R_{b}^{w}{\left[t_{s}^{b}\right]}_{\times }}{\alpha }+\frac{\beta \;n^{⊤}R_{b}^{w}{\left[u^{b}\right]}_{\times }}{\alpha^{2}}\right)\; & \;0_{1\times 3}\;\; & \;0_{1\times 3}\; \end{array}\right].$$

The TFmini altitude measurement directly constrains position (along n) and is sensitive to attitude errors through the lever arm $t_{s}^{b}$ and the beam direction $u^{b}$; it is insensitive to velocity and IMU biases (the corresponding columns are zero). In practice, the ground is often approximated as horizontal and the beam as approximately pointing vertically downward, i.e.,

(40) $$n={\left[0,0,1\right]}^{⊤},\;d=0,\;u^{b}\approx {\left[0,0,-1\right]}^{⊤}.$$

For convenience, let $e_{z}={\left[0,0,1\right]}^{⊤}$ denote the vertical unit vector in the world frame. Then

(41) $$\alpha \approx e_{z}^{⊤}R_{b}^{w}e_{z},\;\beta \approx e_{z}^{⊤}\left(p_{b}^{w}+R_{b}^{w}t_{s}^{b}\right).$$

To reduce complexity, neglecting attitude effects yields a simpler Jacobian approximation:

(42) $$H_{\mathrm{TF}}\approx \left[\begin{array}{ccccc} -\frac{e_{z}^{⊤}}{\alpha }\; & \;0\;\; & \;0\;\; & \;0\;\; & \;0\; \end{array}\right].$$

#### 2.2.4. Filter Update and Error Injection

In the update stage, the observations from UWB, VIO, and TFmini are written in a unified measurement equation:

(43) z=h(x)+n,n∼N(0,R),

where z is the sensor measurement vector, h(·) is the measurement function, and R is the measurement noise covariance. Based on the nominal state $\bar{x}$, the predicted measurement is $\hat{z}=h\left(\bar{x}\right)$, and the residual is computed as

(44) $$r=z-\hat{z}.$$

Using the ROS timestamps, all measurement residuals are interpolated to the UWB measurement times and then stacked as

(45) $$r=\left[\begin{array}{c} r_{\mathrm{uwb}}\\ r_{\mathrm{vio}}\\ r_{\mathrm{tf}} \end{array}\right],\;H=\left[\begin{array}{c} H_{\mathrm{uwb}}\\ H_{\mathrm{vio}}\\ H_{\mathrm{tf}} \end{array}\right],\;R=\mathrm{blkdiag}\;\left(R_{\mathrm{uwb}},R_{\mathrm{vio}},R_{\mathrm{tf}}\right),$$

where H is the measurement Jacobian, with row blocks corresponding to the linearization of different sensors. UWB measurements are sensitive mainly to position, VIO is sensitive to position and attitude, and TFmini is primarily sensitive to altitude and attitude; thus H exhibits a sparse structure.

According to the ESKF update equations, the Kalman gain is computed as

(46) $$K=PH^{⊤}{\left(HPH^{⊤}+R\right)}^{-1}.$$

Using this gain, the error state is updated to obtain the minimum mean-square estimate:

(47) $$\delta \hat{x}=Kr,$$

where r is the measurement residual. Physically, K reflects the balance between the prior uncertainty P and the measurement reliability R.

Meanwhile, the error-state covariance must be updated to reflect the new uncertainty level:

(48) $$P\leftarrow \left(I-KH\right)P{\left(I-KH\right)}^{⊤}+KRK^{⊤}.$$

In practice, a reduced form is often used

(49) $$P\leftarrow \left(I-KH\right)P$$

to reduce computational complexity and improve numerical stability.

Next, the estimated error is injected into the nominal state:

(50) $$\begin{array}{cc} p & \leftarrow p+\delta \hat{p}, \end{array}$$

(51) $$\begin{array}{cc} v & \leftarrow v+\delta \hat{v}, \end{array}$$

(52) $$\begin{array}{cc} q & \leftarrow q⊗\exp\;\left(\frac{1}{2}\delta \hat{\theta }\right), \end{array}$$

(53) $$\begin{array}{cc} b_{a} & \leftarrow b_{a}+\delta {\hat{b}}_{a},\;b_{g}\leftarrow b_{g}+\delta {\hat{b}}_{g}, \end{array}$$

where the attitude is updated by right-multiplying a perturbation; exp(·) denotes the exponential map. Unlike direct additive updates on Euler angles, this approach avoids singularities and preserves the unit-norm constraint of the quaternion.

To re-define the error state as zero-mean after the update, an error reset is performed. The covariance must be corrected using the reset Jacobian $G_{r}$:

(54) $$P\leftarrow G_{r}PG_{r}^{⊤}.$$

Here, $G_{r}$ equals the identity for the position, velocity, and bias blocks, while for the attitude block it is approximated by $I-\frac{1}{2}{\left[\delta \hat{\theta }\right]}_{\times }$, reflecting the nonlinearity of the special orthogonal group.

## 3. Experimental Validation

**Figure 3:**
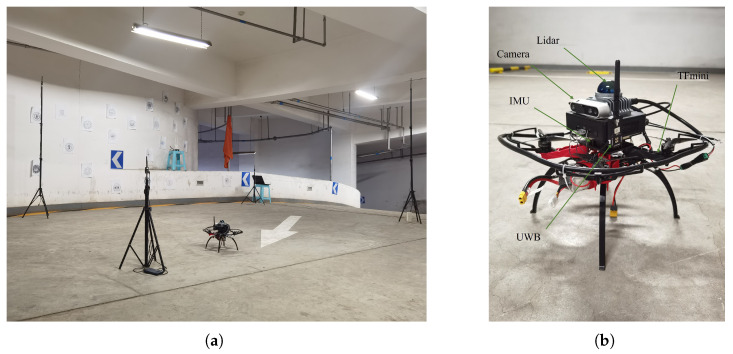
Underground parking garage experiment and platform: (**a**) experimental scene; (**b**) UAV platform.

We built a UAV platform and conducted multiple experiments in an underground parking garage. The experimental area measured approximately 7m×7m×3m, and the overall setup is shown in Figure 3. The UAV was equipped with an IMU (TDK InvenSense Inc., San Jose, CA, USA), a UWB ranging module (Shenzhen Nooploop Technology Co., Ltd., Shenzhen, China), a TFmini laser rangefinder (Benewake Beijing Co., Ltd., Beijing, China), a RealSense D435i camera, (Intel Corporation, Santa Clara, CA, USA) and a MID360 LiDAR (Livox Technology Co., Ltd., Shenzhen, China). Data acquisition and processing were performed onboard by an Intel NUC computer (Intel i5-8400 CPU @ 2.80 GHz, 16 GB RAM, Santa Clara, CA, USA). The sensor configurations were as follows: the UWB module (Nooploop LinkTrack P) measured distances from the tag to anchors at 10 Hz; the IMU (ICM-42688-P) output accelerations and angular velocities at 50 Hz; Visual–Inertial Odometry (VIO) observations were generated using the VINS-Fusion algorithm at 15 Hz; the LiDAR ran the Fast-LIO2 algorithm [43] to provide real-time poses at 20 Hz and served as the system ground truth for accuracy evaluation; the TFmini measured UAV altitude at 50 Hz. Since the extrinsic parameters of the UWB and TFmini have minimal impact on position estimation, their mounting offsets can be neglected in practical applications. Four UWB anchors were deployed inside the experimental area, located at its four corners, with coordinates $p_{A_{1}}^{w}={\left(0,\;0,\;0.81\right)}^{⊤}$, $p_{A_{2}}^{w}={\left(0,\;-4.654,\;0\right)}^{⊤}$, $p_{A_{3}}^{w}={\left(3.823,\;-4.795,\;0.856\right)}^{⊤}$, and $p_{A_{4}}^{w}={\left(3.81,\;0,\;2.211\right)}^{⊤}$.

### 3.1. Comparative Experiments

To rigorously validate the effectiveness and robustness of the proposed algorithm, we conducted multiple comparative experiments in real-world environments. Under identical hardware platforms, scenes, and motion paths, we evaluated seven representative localization methods: Linear Least Squares (LS), Nonlinear Least Squares (NLS), Particle Filter (PF), Loosely Coupled Extended Kalman Filter (EKF-LC), Loosely Coupled ESKF (ESKF-LC), VIO, and the proposed Tightly Coupled ESKF (ESKF-TC). To ensure fairness and reproducibility, all methods were run independently multiple times on the same dataset. The experimental design included five canonical trajectories: circular, rectangular, square, triangular, and rhombic. All trajectories commenced from the same initial position and attitude, followed predetermined paths, and ended with landing at the final waypoint; the flight altitude was approximately constant throughout. During the experiments, sensor data were recorded uniformly via ROS, and data processing and algorithm execution were performed offline. For evaluation, we used the high-accuracy trajectory generated by LiDAR odometry as ground truth (LIO-GT) and employed the Absolute Position Error (APE) to quantitatively compare localization accuracy and stability across methods. Due to space limitations, we present and analyze only the rectangular and circular trajectories; the quantitative metrics and trends for the remaining three trajectories (square, triangular, rhombic) are consistent with those reported.

**Figure 4:**
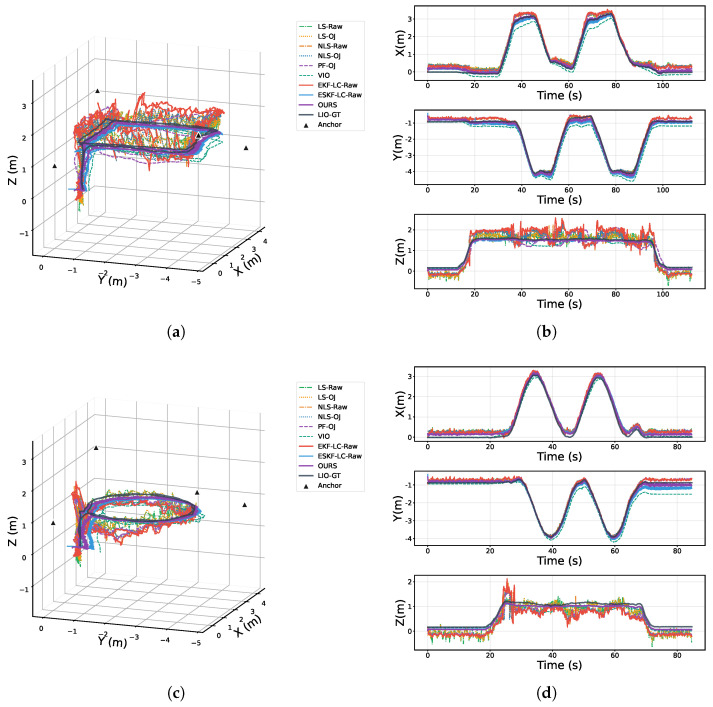
3D localization trajectories and axis-wise position curves for different algorithms: (**a**) rectangular trajectory; (**b**) axis-wise position curves of the circular trajectory; (**c**) circular trajectory; (**d**) axis-wise position curves of the circular trajectory.

Figure 4 presents a comparison of 3D localization trajectories and axis-wise position–time curves for all methods. Specifically, Figure 4a,c show the 3D trajectories for the rectangular and circular paths, respectively, while Figure 4b,d provide the corresponding axis-wise position–time plots. For clarity, different colors and line styles are used to distinguish methods: *Raw* denotes results without outlier rejection, *OJ* denotes results with outlier rejection, and LIO-GT denotes the Fast-LIO2 result, which serves as the ground truth in this paper. From the 3D trajectory comparisons, OURS (ESKF-TC) is overall the closest to LIO-GT, maintaining good agreement even at turning points and during intervals with large velocity changes. In contrast, PF-OJ deviates the most from LIO-GT, exhibiting pronounced cumulative drift. Further inspection of the axis-wise curves in Figure 4b reveals that fluctuations along the (z) axis are generally larger than those along the (x/y) axes across methods, indicating that vertical errors have a more significant impact on overall localization accuracy. By comparison, OURS exhibits lower instantaneous fluctuations and long-term drift on all three axes; in particular, along the (z) axis, thanks to TFmini’s direct altitude constraint and the tightly coupled modeling that exploits measurement consistency, error accumulation and fluctuations are markedly suppressed. Overall, the proposed ESKF-TC demonstrates higher accuracy and robustness through multi-source fusion (UWB+VIO+TFmini).

**Figure 5:**
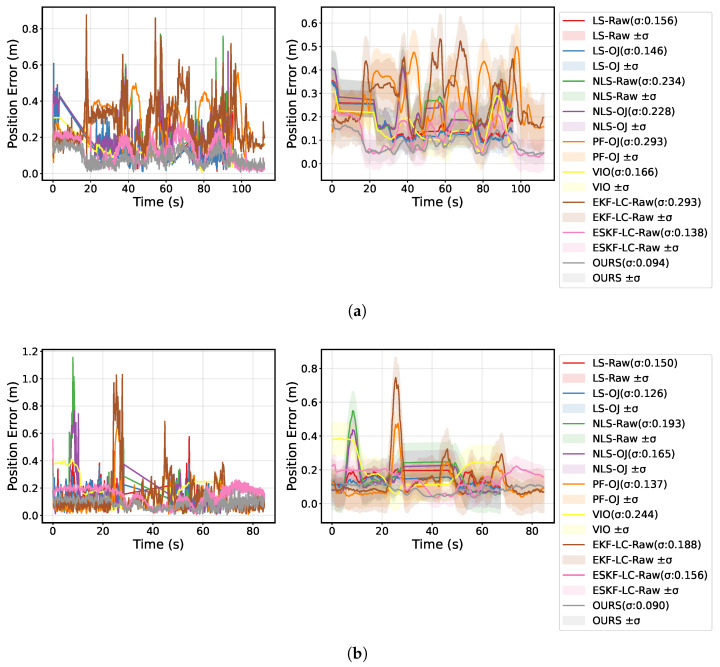
Localization error comparison for different trajectories: (**a**) rectangular trajectory localization error curve; (**b**) circular trajectory localization error curve.

Figure 5 shows the absolute position error (APE) curves over time for each algorithm, along with the smoothed error (moving average) and its relationship with time, including the 1σ confidence bounds. From the figure, it can be seen that OURS maintains the lowest error level throughout, with its 1σ confidence interval significantly converging and being the narrowest. These results indicate that OURS, when fusing UWB, VIO, and TFmini observations, achieves higher accuracy and stability, effectively suppressing error accumulation and short-term fluctuations.

**Figure 6:**
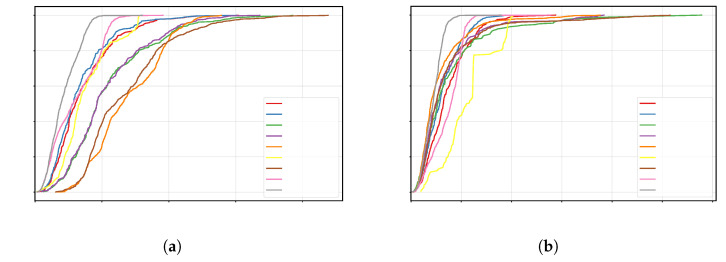
CDF localization error comparison for different algorithms and trajectories: (**a**) rectangular trajectory CDF error curve; (**b**) circular trajectory CDF error curve.

Figure 6 shows the cumulative distribution function (CDF) of the APE for each algorithm: the horizontal axis represents the error threshold, and the vertical axis represents the probability of error not exceeding that threshold. Generally, the curve positioned further to the left and rising faster indicates smaller errors at most times and higher overall accuracy. From the figure, it can be seen that the CDF curve of OURS is consistently positioned to the upper-left of the other methods for both trajectory types, reflecting a significant accuracy advantage. For quantitative comparison, we use the 68.3% quantile error (approximately 1σ) as the evaluation metric: for the circular trajectory, the error for OURS is 0.1077m, better than the second-best PF-OJ (0.1182m), representing an approximately 8.9% reduction; for the rectangular trajectory, OURS has an error of 0.1076m, better than the second-best LS-OJ (0.1501m), representing an approximately 28.3% reduction. Furthermore, VIO and several loosely coupled/single-observation variants exhibit significantly larger 1σ position errors (e.g., for VIO on the circular trajectory, 0.2473m, and for ESKF-LC-Raw on the rectangular trajectory, 0.1699m), indicating that missing other sensor observations or failing to suppress outliers limits both the leftward shift and steepness of the distribution. In summary, OURS shifts the error distribution leftward, significantly improving steepness, achieving lower typical errors, and demonstrating stronger robustness across different trajectory shapes.

As shown in Table 1, we systematically compare the APE of each algorithm on two trajectory types (Square and Circular) using RMSE, Mean, Std, Min, Max, and Median. Overall, OURS achieves the lowest RMSE, Mean, and Std on both trajectories, and is also best on Min and Median for the Square trajectory, as well as Max for the Circular trajectory. The few exceptions are as follows: the Square-Max is achieved by VIO with a value of 0.3174; the Circular-Min is achieved by ESKF-LC-Raw with 0.0035; and the Circular-Median is achieved by PF-OJ with 0.0786. In terms of magnitude, for the Square trajectory, OURS reduces RMSE from 0.1402 to 0.0972 relative to the second-best baseline ESKF-LC-Raw (a reduction of about 30.7%), Mean from 0.1212 to 0.0864 (about 28.7%), and Std from 0.0704 to 0.0446 (about 36.7%). For the Circular trajectory, OURS reduces RMSE from 0.1434 to 0.0944 relative to the best baseline LS-OJ (about 34.2%), Mean from 0.1246 to 0.0863 (about 30.7%), and reduces Std from 0.0624 to 0.0382 relative to ESKF-LC-Raw (about 38.8%). Although certain methods are superior on a single statistic (e.g., VIO for Square-Max, ESKF-LC-Raw for Circular-Min, PF-OJ for Circular-Median), their overall error levels and dispersion are clearly worse than those of OURS. These results indicate that introducing outlier rejection and tightly coupled ESKF fusion of multi-source observations effectively improves measurement quality and fusion stability, thereby achieving higher localization accuracy and robustness across different motion trajectories.

### 3.2. Ablation Study

To verify the effectiveness of the key modules in the proposed algorithmic framework and to assess the impact of potential module failures [44], we conducted an ablation study for comparative analysis. The evaluated variants include: a tightly coupled fusion method without UWB outlier rejection but fusing VIO and TFmini (TC Both-RAW); a tightly coupled method with outlier rejection but without the VIO position constraint (TC TFmini-OJ); a tightly coupled method with outlier rejection but without the TFmini altitude constraint (TC VIO-OJ); and a tightly coupled UWB–IMU fusion method with outlier rejection (TC UWB-OJ). Finally, we compare all variants against the proposed tightly coupled fusion method integrating IMU, UWB, VIO, and TFmini (OURS). All methods were tested through multiple independent trials under identical experimental environments, sensor configurations, and motion trajectories. Except for the ablated modules, the remaining implementation details and parameter settings were kept consistent to ensure fair comparison.

**Figure 7:**
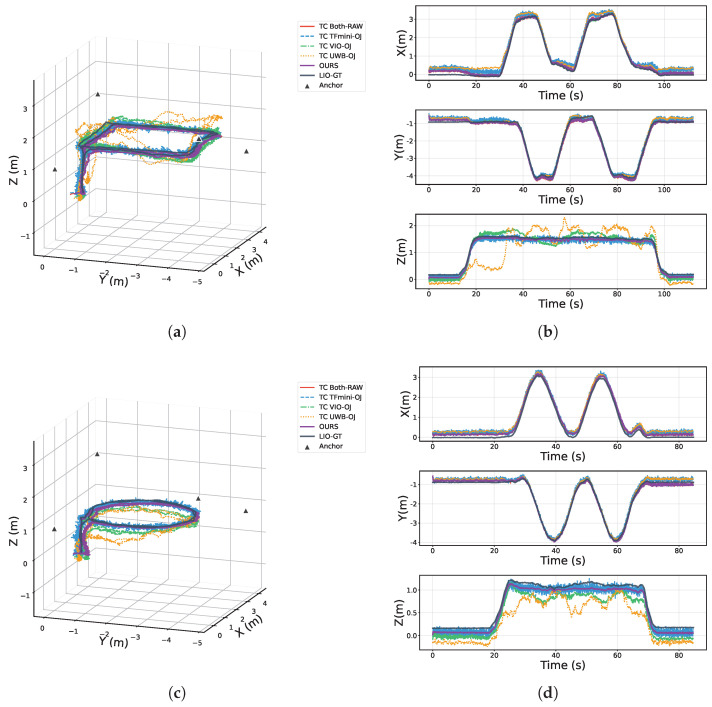
3D localization trajectories and axis-wise position curves for different algorithms: (**a**) rectangular trajectory; (**b**) axis-wise position curves of the circular trajectory; (**c**) circular trajectory; (**d**) axis-wise position curves of the circular trajectory.

Figure 7 compares the 3D localization trajectories and the axis-wise position–time curves of all methods. Specifically, Figure 7a and Figure 7c show the 3D trajectories for the rectangular and circular paths, respectively, while Figure 7b and Figure 7d present the corresponding axis-wise position–time curves. It can be observed that OURS achieves the best localization accuracy among all methods in the ablation study, with trajectories closest to the LiDAR ground truth (LIO-GT). In contrast, removing the TFmini altitude constraint or the RANSAC outlier rejection module leads to noticeably larger deviations from ground truth, especially in regions with altitude changes or where UWB outliers are present, resulting in more pronounced error fluctuations. Moreover, methods that rely solely on UWB or VIO produce trajectories with substantial deviations and exhibit significant instability and drift. Overall, by means of tightly coupled multi-sensor fusion and robust outlier handling, OURS effectively improves localization accuracy and robustness, enabling high-precision UAV localization in complex environments.

**Figure 8:**
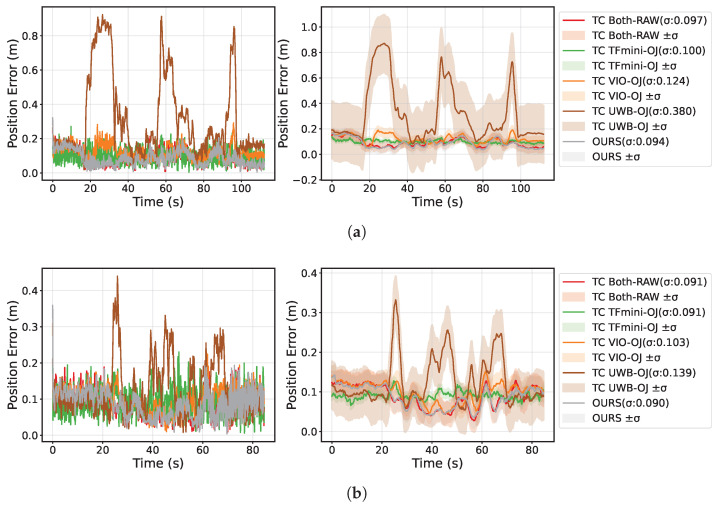
Localization error comparison for different trajectories: (**a**) rectangular trajectory localization error curve; (**b**) circular trajectory localization error curve.

Figure 8 presents the 3D position error profiles for all methods on both the rectangular and circular trajectory experiments. For each trajectory, the left subfigure plots the instantaneous absolute position error (i.e., the Euclidean distance between the estimated position and the ground truth) as a function of time, while the right subfigure shows the corresponding temporally smoothed error curve together with the 1σ band computed over the entire trajectory. As seen in the figure, OURS exhibits the lowest error curve and the narrowest confidence interval among all ablation variants, indicating superior accuracy and stability. In comparison, removing the TFmini altitude constraint or the RANSAC outlier rejection module raises the overall error level and enlarges the fluctuation range, with more pronounced peaks in segments featuring altitude changes or UWB outliers. Additionally, methods relying solely on UWB or VIO show large fluctuations and strong instability and drift. Overall, through tightly coupled fusion and robust outlier rejection, OURS effectively suppresses error accumulation and abrupt changes, significantly enhancing localization accuracy and robustness.

**Figure 9:**
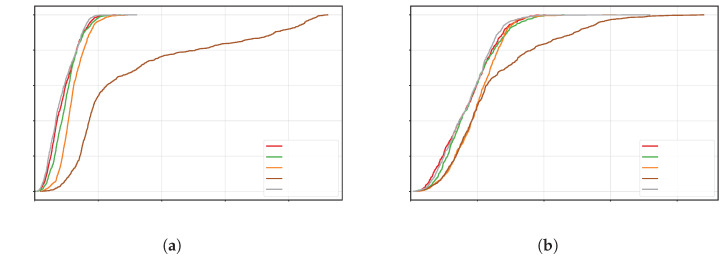
CDF localization error comparison for different algorithms and trajectories: (**a**) rectangular trajectory CDF error curve; (**b**) circular trajectory CDF error curve.

Figure 9 presents the CDF of absolute errors for each method. In the ablation study, the CDF curve of OURS consistently lies to the upper-left of the other methods, indicating lower absolute position errors across all trajectory shapes. Quantitatively, at the 1σ position (approximately the 68% quantile), OURS attains threshold errors of 0.1076 and 0.1077 for the rectangular and circular trajectories, respectively—the smallest among all methods; in contrast, TC UWB-OJ yields the largest thresholds, 0.3114 (rectangular) and 0.1342 (circular). Aggregating statistics across different trajectory shapes, OURS achieves smaller errors for the vast majority of time, significantly outperforming the other ablation variants and reflecting higher accuracy. These results demonstrate that OURS, via tightly coupled multi-sensor fusion and robust outlier rejection, effectively improves localization accuracy and stability, enabling high-precision UAV localization in complex environments.

As shown in Table 2, we compare the APE statistics of all methods on two trajectory types (Square and Circular). OURS achieves the lowest RMSE and Mean on both trajectories and also the smallest Median; its Std is near-optimal (Square: 0.0446, Circular: 0.0382), slightly higher than TC VIO-OJ (0.0411/0.0362), indicating a low overall dispersion of errors. Although TC Both-RAW yields smaller maximum errors in the Max metric (Square: 0.2560; Circular: 0.2019), its RMSE/Mean is overall worse than OURS due to its susceptibility to abnormal UWB ranges without outlier rejection. The TC UWB-OJ (with outlier rejection enabled) exhibits notably larger RMSE and Std on both trajectories, indicating difficulty in suppressing cumulative drift and fluctuations without visual and altitude constraints. Variants that remove a single observation, TC TFmini-OJ and TC VIO-OJ, may be better on individual statistics (e.g., Min/Std), but their overall accuracy and stability remain inferior to OURS. In terms of quantitative gains, relative to TC Both-RAW (no outlier rejection), OURS reduces RMSE by about 4.0%/1.1% on Square/Circular trajectories; relative to TC TFmini-OJ/TC VIO-OJ, OURS improves RMSE by about 10.2%/23.4% (Square) and 4.6%/11.2% (Circular). These results indicate that multi-epoch outlier rejection and tightly coupled ESKF fusion of IMU/UWB/VIO/TFmini effectively enhance measurement quality and fusion stability, significantly reducing localization errors and their dispersion.

## 4. Conclusions

This paper targets GNSS-denied, heavily occluded indoor environments and proposes a tightly coupled multi-sensor localization framework based on the ESKF. The core idea is to drive state prediction with IMU motion model, incorporate raw UWB ranges, VIO relative poses, and TFmini altitude jointly in the measurement update, and apply a VIO-constrained multi-epoch outlier rejection to perform geometric consistency screening of UWB measurements, thereby suppressing NLOS-induced outliers at the source. Real-world experiments in an underground parking garage show that, on rectangular and circular trajectories, the proposed method attains the best performance on typical metrics (Square: 0.0972/0.0864/0.0446 for RMSE/Mean/Std; Circular: 0.0944/0.0863/0.0382), with CDF error thresholds at the 68.3% quantile of 0.1076/0.1077 m, and curves that are overall “more left and steeper,” indicating smaller errors and stronger stability for the vast majority of time. Ablation results further validate the necessity of each module: removing the altitude constraint or outlier rejection yields noticeably larger errors and fluctuations; relying only on IMU-UWB leads to more pronounced cumulative drift. Overall, the synergy of tightly coupled fusion and robust preprocessing enables high-frequency, accurate, and robust localization under multi-source asynchrony, multipath interference, and locally degraded conditions.

## Figures and Tables

**Table 1:** Localization Error Statistics Comparison of Different Algorithms on Various Trajectories.

Methods	Square Trajectory						Circular Trajectory					
	RMSE	Mean	Sth	Min	Max	Median	RMSE	Mean	Sth	Min	Max	Median
LS-Raw	0.1736	0.1469	0.0926	0.0127	0.6076	0.1209	0.1709	0.1467	0.0878	0.0097	0.5756	0.1290
LS-OJ	0.1617	0.1357	0.0880	0.0103	0.6019	0.1142	0.1434	0.1246	0.0710	0.0147	0.3764	0.1144
NLS-Raw	0.2584	0.2228	0.1309	0.0259	0.7701	0.1856	0.2268	0.1576	0.1631	0.0114	1.1560	0.1068
NLS-OJ	0.2468	0.2163	0.1188	0.0201	0.6720	0.1851	0.1915	0.1392	0.1315	0.0125	0.7675	0.1016
PF-OJ	0.2967	0.2774	0.1053	0.0615	0.5569	0.2604	0.1547	0.1149	0.1036	0.0080	0.7522	**0.0786**
VIO	0.1692	0.1519	0.0746	0.0058	**0.3174**	0.1335	0.2452	0.2239	0.0999	0.0396	0.4108	0.2270
EKF-LC-Raw	0.3042	0.2744	0.1313	0.0627	0.8757	0.2397	0.2097	0.1453	0.1512	0.0117	1.0306	0.0991
ESKF-LC-Raw	0.1402	0.1212	0.0704	0.0078	0.3823	0.1132	0.1585	0.1457	0.0624	**0.0035**	0.5564	0.1595
**OURS**	**0.0972**	**0.0864**	**0.0446**	**0.0056**	0.3207	**0.0779**	**0.0944**	**0.0863**	**0.0382**	0.0041	**0.3588**	0.0871

Note: All values are in meters (m). RMSE = Root Mean Square Error, Std = Standard Deviation. Bold values indicate the best performance in each metric.

**Table 2:** Ablation Study: Localization Error Statistics Comparison on Different Trajectories.

Methods	Square Trajectory						Circular Trajectory					
	RMSE	Mean	Std	Min	Max	Median	RMSE	Mean	Std	Min	Max	Median
TC Both-RAW	0.1013	0.0910	0.0445	0.0086	**0.2560**	0.0849	0.0955	0.0869	0.0397	0.0095	**0.2019**	0.0880
TC TFmini-OJ	0.1082	0.0995	0.0425	**0.0039**	0.2713	0.0968	0.0990	0.0907	0.0396	0.0061	0.2304	0.0878
TC VIO-OJ	0.1269	0.1201	**0.0411**	0.0246	0.2973	0.1148	0.1063	0.1000	**0.0362**	0.0099	0.3081	0.1006
TC UWB-OJ	0.3884	0.3063	0.2388	0.0191	0.9226	0.1878	0.1460	0.1252	0.0752	0.0078	0.4397	0.1017
**OURS**	**0.0972**	**0.0864**	0.0446	0.0056	0.3207	**0.0779**	**0.0944**	**0.0863**	0.0382	**0.0041**	0.3588	**0.0871**

Note: All values are in meters (m). RMSE = Root Mean Square Error, Std = Standard Deviation. Bold values indicate the minimum in each column.

## Data Availability

The original contributions presented in this study are included in the article. Further inquiries can be directed to the corresponding author(s).
